# Association between spirochaetal infection and neurodegenerative diseases: a systematic review and quantitative synthesis of observational studies

**DOI:** 10.1099/jmm.0.002136

**Published:** 2026-03-06

**Authors:** Mia Horton, Daniel J. Whiley, Megan Mayhew, Samantha McLean

**Affiliations:** 1 School of Science and Technology, Nottingham Trent University, Nottingham, UK; 2 Medical Technologies Innovation Facility, Nottingham Trent University, Nottingham, UK

**Keywords:** *Borrelia burgdorferi*, *Leptospira* spp., neurodegenerative disease, spirochaete, systematic review, *Treponema pallidum*

## Abstract

**Introduction.** Neurodegenerative diseases, including Alzheimer’s and Parkinson’s, are a growing global health concern. While age remains the primary risk factor, infectious agents have been proposed as potential contributors to disease onset or progression.

**Gap statement.** Spirochaetal bacteria, such as *Treponema pallidum*, *Borrelia burgdorferi* and *Leptospira* spp., can invade the central nervous system, yet the extent to which these infections influence neurodegenerative outcomes remains unclear.

**Aim.** This systematic review aimed to evaluate observational evidence on the association between spirochaetal infections and neurodegenerative diseases and to identify gaps in the literature to inform future research.

**Methodology.** A systematic search of SCOPUS, EMBASE, PubMed/MEDLINE, Web of Science and CINAHL was conducted for studies published between January 2000 and May 2025. Eligible studies were observational, involved adult human populations and reported both spirochaetal infection and cognitive or neurodegenerative outcomes using standardized methods. Data were extracted using a standardized form. Owing to heterogeneity in study design, diagnostic approaches, outcome measures and reporting formats, an overall pooled meta-analysis was not feasible; however, a quantitative synthesis using meta-analytic methods was conducted for studies reporting mini-mental state examination data. Risk of bias was assessed using the Newcastle–Ottawa Scale.

**Results.** Twenty-seven studies met the inclusion criteria: 13 on *T. pallidum*, 13 on *B. burgdorferi* and one on *Leptospira* spp. No eligible studies were found for *Brachyspira* spp., and studies involving *Treponema denticola* were excluded due to confounding by periodontitis. Studies investigating syphilis and leptospirosis consistently reported cognitive impairment and increased dementia risk. In contrast, findings for Lyme disease were heterogeneous, with some studies reporting persistent symptoms or increased Alzheimer’s risk, while others found no long-term cognitive effects.

**Conclusion.** This review highlights a potential link between spirochaetal infections and neurodegenerative outcomes, particularly for syphilis and leptospirosis. Evidence for Lyme disease remains inconclusive. Future research should prioritize longitudinal studies with standardized diagnostic criteria, integration of neuroimaging and biomarker data and improved diagnostic accuracy for spirochaetal infections.

## Introduction

Neurodegenerative diseases are characterized by the progressive loss of neuronal structure and function, collectively referred to as neurodegeneration. This umbrella term encompasses a range of conditions, including dementia-related disorders, demyelinating diseases, parkinsonian syndromes and motor neuron diseases [1]. According to the World Health Organization, an estimated 57 million people were living with dementia globally in 2021, a figure projected to rise to 75 million by 2030 and 132 million by 2050 [2]. Alzheimer’s disease is the most prevalent form, accounting for ~60–70 % of cases. The societal and economic burden of neurodegenerative diseases is substantial; in the UK alone, the cost of dementia was estimated at £42.5 billion in 2024, with projections suggesting this will at least double by 2040 [3]. Age remains the most significant risk factor for neurodegenerative diseases. As global populations age, it is increasingly important to identify causal or exacerbating factors that may contribute to disease onset or progression to mitigate both personal and economic impacts.

One emerging area of investigation is the potential role of infectious agents in the pathophysiology of neurodegenerative diseases. A seminal study published in 1997 identified latent herpes simplex virus type 1 in the brains of elderly individuals with Alzheimer’s disease [4]. Since then, the infectious hypothesis has expanded to include a variety of bacterial, viral and fungal pathogens, such as the dental pathogen *Porphyromonas gingivalis* and the respiratory tract pathogen *Chlamydia pneumoniae* [5,6]. In parallel, chronic neuroinflammation and sustained immune activation have been recognized as central features of neurodegenerative pathology [7]. For example, systematic reviews have identified a significant association between periodontitis and neurodegenerative disease, including higher levels of inflammatory markers; however, causality cannot be inferred due to the methodological heterogeneity of included studies [8,9].

Spirochaetes warrant particular attention among candidate infectious drivers of neurodegeneration because they are proven neurotropic pathogens with documented capacity for central nervous system invasion, immune evasion and persistence. Late-stage *Treponema pallidum* infection (neurosyphilis) represents a well-established example, with clear mechanistic evidence of blood–brain barrier traversal and neuroinflammatory injury [10,11]. In contrast, proposed links between *Borrelia burgdorferi* and neurodegenerative disease remain debated. While *B. burgdorferi* DNA and antigens have been detected in human brain tissue and in some reports colocalize with amyloid markers, raising biologically plausible links to Alzheimer’s pathology [12,13], epidemiological studies have not consistently demonstrated an association between Lyme disease incidence and major neurodegenerative disorders [14]. Similar uncertainty has been applied to other spirochaetes, such as *Treponema denticola*, a periodontal spirochaete, where animal models have shown neuroinflammatory changes, but human evidence is limited and heterogeneous [8,9, 15, 16]. These shared traits, motility, tissue invasiveness and chronicity, distinguish spirochaetes from many other pathogens implicated in the infectious hypothesis and provide rationale for a focussed synthesis of the available evidence.

A comprehensive and systematic review of the associations between spirochaetal infection and neurodegenerative diseases does not exist within the current literature. This systematic review aims to consolidate existing observational evidence on the association between spirochaetal infection and neurodegenerative disease. A secondary objective is to identify gaps in the current literature and highlight areas for future research into the pathogenic mechanisms of neurotropic spirochaetes, to inform evidence-based approaches to diagnosis and treatment.

## Methods

### Protocol development and registration

The study protocol was developed in accordance with the Preferred Reporting Items for Systematic Reviews and Meta-Analyses (PRISMA) guidelines (Table S1, available in the online Supplementary Material) [17] and registered with the International Prospective Register of Systematic Reviews (PROSPERO) under the registration number CRD420251014990 [18].

### Study eligibility criteria

Studies were eligible for inclusion if they met the following criteria (1): published, peer-reviewed original articles (2); human studies conducted in adult populations (3); diagnosis or seropositivity of spirochaetal infection reported to a clinically acceptable standard (4); methods for assessment of cognitive function, impairment or decline, including diagnoses of Alzheimer’s disease, Parkinson’s disease or other dementias present (5); observational study design (cross-sectional, cohort or case–control; prospective or retrospective) and (6) studies written in English with full text availability.

Studies were excluded according to the following criteria (1): reviews, letters, commentaries, grey literature, editorials, case reports, conference papers and surveys (2); the use of non-standardized or self-reported criteria to define cognitive impairment or infection status and (3) interventional studies, including randomized control trials.

For this review, ‘clinically acceptable’ or ‘standardized’ diagnostic criteria for spirochaetal infection were defined as the use of validated laboratory methods appropriate to each organism. These included for *T. pallidum*: treponemal (e.g. *T. pallidum* particle agglutination/haemagglutination assay and fluorescent treponemal antibody absorption test) and non-treponemal (e.g. rapid plasma reagin test and venereal disease research laboratory test) tests used in accordance with clinical guidelines. For *B. burgdorferi*, diagnosis utilized validated two-tier or modified two-tier serological testing or cerebrospinal fluid (CSF) antibody index, where applicable. Seropositivity was defined as either a positive ELISA or a positive or equivocal Western blot for *Leptospira* spp.: ELISA, microscopic agglutination test or PCR-based confirmation.

Cognitive impairment was considered standardized when assessed using validated tools [e.g. Mini-Mental State Examination (MMSE), Montreal Cognitive Assessment (MoCA) and Clinical Dementia Rating scale] or formal diagnostic criteria [e.g. Diagnostic and Statistical Manual of Mental Disorders (DSM-5 [19]), International Classification of Diseases (ICD-11 [20]) and NINCDS–ADRDA Alzheimer’s Diagnostic Criteria [21]]. Studies relying on self-reported infection status, non-validated assays or unstandardized cognitive measures were classified as non-standardized and excluded.

Studies investigating *T. denticola* were excluded because the presence of periodontitis in all publications introduces substantial confounding, making it difficult to attribute cognitive or neurodegenerative outcomes specifically to spirochaetal infection.

### Data sources and search strategy

Preliminary scoping searches were conducted to refine the search terms prior to the formal electronic search on 6 May 2025. The following databases were systematically searched: SCOPUS, EMBASE, PubMed/MEDLINE, Web of Science and CINAHL, covering the period from 1 January 2000 to 6 May 2025. A consistent search strategy was applied across all databases, with formatting adapted to suit each platform (Table S2). In addition, reference lists and citations of manuscripts identified during the scoping searches were screened to identify any further relevant studies for inclusion.

### Study selection

Titles, authors, publication year and abstracts were exported to Zotero (version 7.0.15), where duplicates were removed using the Zoplicate plugin (version 3.0.8). The remaining records were imported into Rayyan for screening [22]. Two independent reviewers screened titles and abstracts against the eligibility criteria (M.H. and D.J.W.). Discrepancies were resolved through discussion and, where necessary, by consultation with a third reviewer (M.M.). Full-text articles of potentially eligible studies were then independently assessed for relevance and inclusion (M.H. and D.J.W.). Where multiple publications appeared to report on the same dataset, the most recent or comprehensive study was retained to avoid duplication and overestimation of findings.

### Data extraction, synthesis and analysis

Following full-text screening, data were extracted using a standardised Excel form by three independent reviewers (M.H., D.J.W. and M.M., Table S9). Where data were missing, this was recorded as ‘not stated’ and considered during the risk of bias assessment. Data were collected across three domains: (1) study characteristics (first author, country of origin, year of publication, funding source, study design, stated aim or objective, sample size, duration of follow-up if applicable and method of participant recruitment); (2) participant characteristics (mean age, gender, number of participants, diagnostic methods including histology and other relevant infection-related data and confounders such as co-medications and co-morbidities) and (3) outcome measures, including reported estimates, cumulative incidence and incidence rates of neurodegenerative disease (e.g. generalized dementia, Alzheimer’s disease, Parkinson’s disease or parkinsonism and multiple sclerosis). Where available, odds ratios, risk ratios and 95 % confidence intervals were extracted. Where quantitative data were not reported, findings were summarized qualitatively.

A quantitative data synthesis using meta-analytic methods was then undertaken for studies reporting MMSE data. Reported means and sample sizes were extracted as reported. Where only minimum and maximum values were available, standard deviations were estimated using established methods for deriving standard deviations from the range and sample size, as recommended for meta-analytic use [23]. Study means were centred on the cognitive impairment threshold (MMSE=23), such that positive values indicated performance above the impairment threshold and negative values indicated impairment. Centred means and their corresponding standard errors were entered into RevMan 5.3 using the generic inverse variance method to generate forest plots. Subgroup summaries were produced for each diagnostic category; however, no overall pooled estimate was calculated owing to substantial clinical and methodological heterogeneity across studies. For all other outcomes, where quantitative synthesis was not feasible due to variability in infection definitions, cognitive measures or reporting formats, findings were synthesized narratively.

### Risk of bias and quality assessment

All included studies were subjected to a methodological quality assessment to evaluate potential sources of bias in study design, conduct and analysis. Two reviewers (M.H. and D.J.W.) independently assessed each study using the Newcastle–Ottawa Scale (NOS) [24], assigning a score from 0 to 9 based on selection, comparability and outcome/exposure domains (Table S3). No studies were excluded based on NOS score; all studies meeting the eligibility criteria were retained regardless of quality rating; all included studies achieved a NOS score of 5–9 (Table S3), which likely reflects the robustness of the inclusion criteria applied.

## Results

### Search results and characterization of studies

**Fig. 1.**
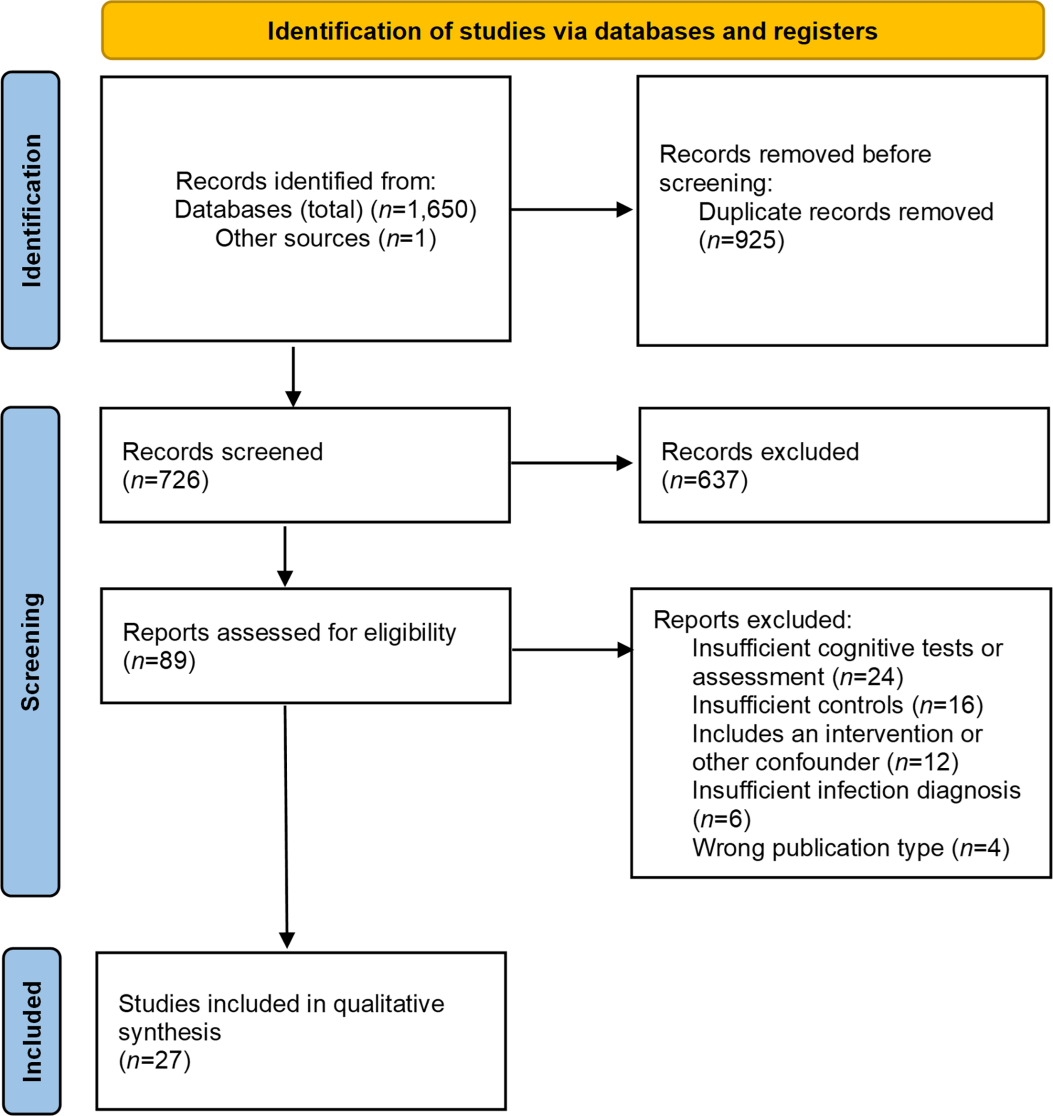
PRISMA flow diagram for the study selection process.

The initial database search yielded 1,650 records, with one additional record identified through reference list screening. After removal of duplicates, 726 unique articles remained. Title and abstract screening, conducted according to the eligibility criteria, resulted in the exclusion of 637 articles. A total of 89 articles were selected for full-text review. Of these, 27 studies met the inclusion criteria and were included in the systematic review. Reasons for exclusion at the full-text stage included insufficient cognitive tests or assessment, including self-reporting or non-standardized measures (*n*=24), inadequate control group, such as the absence of controls or inclusion of controls with spirochaetal infection (*n*=16), inclusion of interventional components or other confounders (*n*=12), insufficient diagnostic criteria for infection (*n*=6) and ineligible publication types (*n*=4). The study selection process is summarized in Fig. 1.

Of the 27 studies examining associations between spirochaetal infections and cognitive decline or neurodegenerative disease, 13 studies investigated *T. pallidum* (syphilis), 13 focused on *B. burgdorferi* (Lyme disease) and 1 examined *Leptospira* spp. (leptospirosis). Findings were synthesized thematically across these pathogens. No eligible studies linked intestinal spirochaetosis (*Brachyspira* spp.) to neurodegenerative disorders. Furthermore, although eight studies included *T. denticola* in their analyses and otherwise met the inclusion criteria, they were excluded due to the presence of periodontitis as a confounding factor [25,32]. In these studies, the comorbidity and involvement of other periodontal pathogens made identification of the role of *T. denticola* in neurodegenerative outcomes difficult.

### Association between *T. pallidum* infection and neurodegenerative diseases

**Fig. 2.**
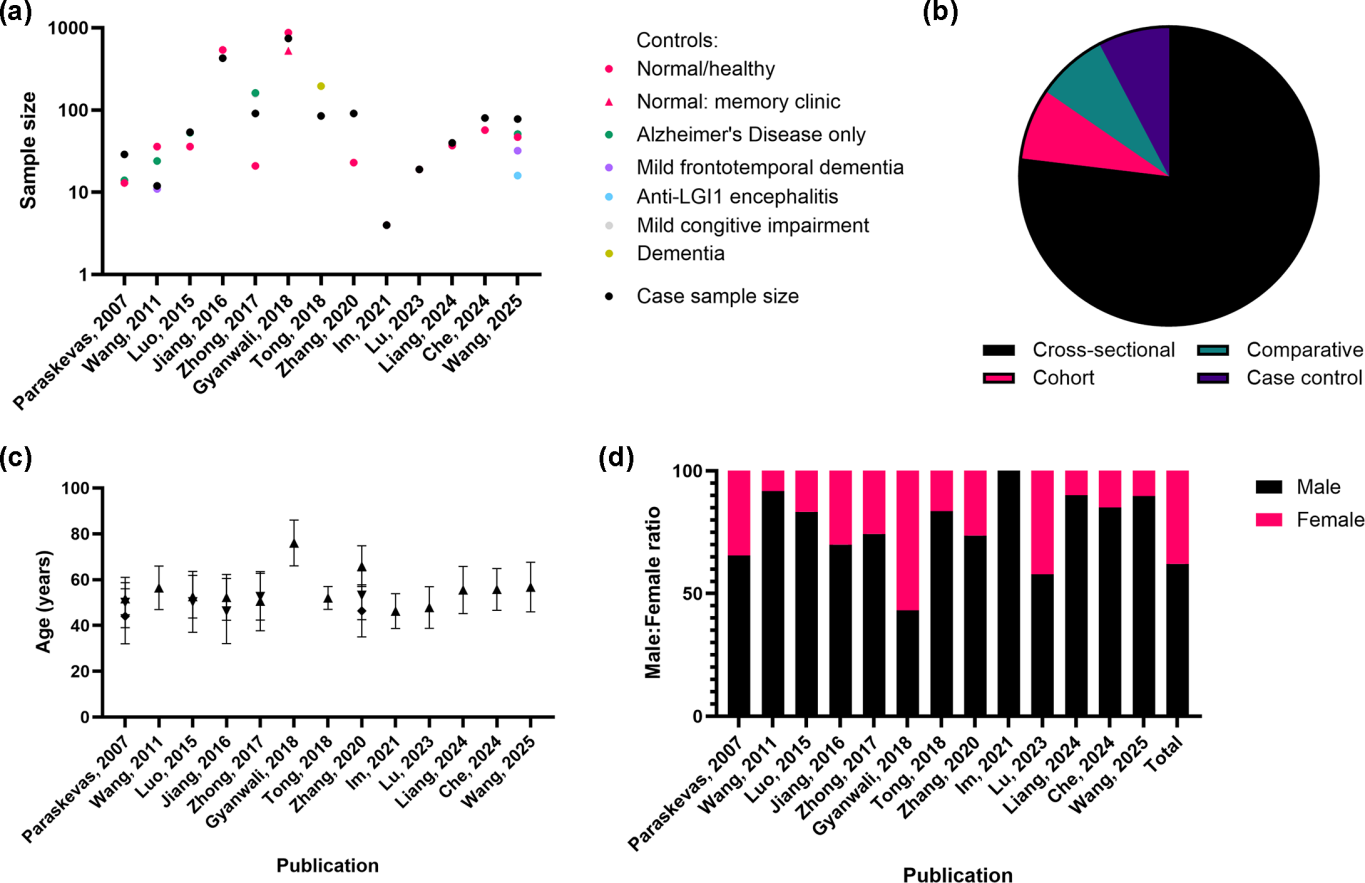
Description of population by study for syphilis. (**a**) Comparison of sample sizes for cases vs. controls, (**b**) number of publications by study design, (**c**) mean age of participants in the studies (±sd) and (**d**) ratio of male to female participants per study.

Thirteen studies investigating the association between *T. pallidum* infection and neurodegenerative disease were included in this review (Fig. 2 and Table S4). Of these, ten studies were conducted in China and primarily focused on clinical comparisons between general paresis and neurosyphilis and their differentiation from Alzheimer’s disease. The methodologies employed across these studies varied and included neuroimaging, biomarker analysis, neuropsychological testing and clinical assessments. These approaches were used to characterize cognitive impairment and neurological outcomes in individuals with late-stage syphilis and to explore potential overlaps with neurodegenerative disease phenotypes.

Central to all studies was the observation of cognitive impairment in individuals with neurosyphilis. Of the 13 studies on * T. pallidum*, 10 reported dementia-level cognitive impairment, with neuroimaging findings including frontal and insular hypoperfusion, grey matter loss and/or whole-brain atrophy. Where tested, biomarker profiles showed elevated tau levels in neurosyphilis patients, though consistently lower than in Alzheimer’s disease [33]. In this context, elevated total tau is interpreted as a marker of neuroaxonal injury rather than a disease-specific biomarker. Across included studies, tau levels in neurosyphilis tended to be higher than in syphilis without central nervous system (CNS) involvement or controls, yet consistently lower than in Alzheimer’s disease, supporting its use for differentiation (neurosyphilis vs. Alzheimer’s disease) rather than implying shared causality.

Four studies used neuroimaging techniques including voxel- and surface-based morphometry, single-photon emission computed tomography (SPECT) and traditional magnetic resonance imaging (MRI) [34,37]. These studies reported findings such as hypoperfusion in the frontal, insular and posterior cingulate regions, regional reductions in grey matter volume and whole-brain atrophy [34,37].

Six studies investigated CSF and metabolic biomarkers in neurosyphilis, including neurogranin, *β*-amyloid precursor protein cleaving enzyme (BACE1), A*β* 1-40 (A*β*40), A*β*42, total tau and metabolic indicators such as cholesterol and apolipoprotein A-I (apoA-I) [33,38,42]. Amongst the three studies assessing CSF total tau levels, all reported significant differences between groups [38,40, 43]. Notably, tau levels were consistently higher in patients with Alzheimer’s disease compared to those with any stage of syphilis infection, although one study found elevated tau levels in patients with general paresis relative to those with syphilis without CNS involvement [33]. In terms of metabolic characteristics, patients with general paresis exhibited reduced *N*-acetylaspartate–creatine and *N*-acetylaspartate–choline ratios in the bilateral hippocampus compared to healthy controls [38]. Additionally, when compared to patients with non-neurosyphilis dementia, significantly different levels of total cholesterol, low-density lipoprotein cholesterol and homocysteine were observed [42].

Three studies focused on the neurocognitive and neuropsychiatric aspects of neurosyphilis. Findings included significant olfactory dysfunction in general paresis patients and distinct neuropsychiatric profiles in neurosyphilis compared to Alzheimer’s disease [41,44]. One study conducted within a memory clinic setting reported a significant association between syphilis seroreactivity and dementia in memory clinic attendees [45]. The objectives, methodologies and key findings of these studies are summarized in Tables 1 and S5.

**Table 1.** Description of objectives, treatments and main outcomes by study (syphilis)

Author	Objective	Treatment	Main outcome	NOS score	Overview
Che *et al*. [38]	To utilize hydrogen proton magnetic resonance spectroscopy to assess metabolite concentrations in GP patients’ bilateral hippocampus	No information	GP patients showed significantly lower *N*-acetylaspartate, creatine and choline ratios in the bilateral hippocampus region when compared to controls. These ratios were not significant in those with mild cognitive impairment when compared to controls. There was no correlation between MMSE scores and metabolite ratios	6	There are significant differences in metabolic characteristics in the hippocampus of GP patients
Gyanwali *et al*. [45]	To assess the prevalence of syphilis reactivity in memory clinic patients and investigate its association with neurodegeneration	No information	There was a significant association between syphilis reactivity and dementia in memory clinic patients, independent of demographic factors (odds ratio: 2.06). There was no significant association between reactivity and any markers of cerebrovascular disease and neurodegeneration	6	Syphilis reactivity and dementia in memory clinic patients are significantly associated
Im *et al*. [37]	To use single-photon emission computed tomography imaging to investigate brain perfusion abnormalities in neurosyphilis patients	Not treated	All cognitive assessment scores indicated cognitive impairment in patients with NS. NS patients showed multifocal hypoperfusion predominantly in the frontal, insular and posterior cingulate regions compared to healthy controls	7	Hypoperfusion in frontal, insular and posterior cingulate regions of the brain may reflect cognitive impairments in NS patients
Jiang *et al*. [39]	To retrospectively investigate the association between lipid metabolism and GP	Penicillin	Significantly lower apolipoprotein A-I levels were found in GP and in syphilitic patients without neurosyphilis compared to controls. The follow-up evaluation of 35 GP patients 3 months after penicillin treatment showed a significant positive correlation between increased apoA-I levels and MMSE scores	5	Abnormal apolipoprotein A-I levels may be associated with a cognitive decline in syphilitic dementia
Liang *et al*. [44]	To investigate whether GP patients experience olfactory dysfunction and assess its relatedness to clinical manifestations	Standardized treatment	Patients experienced significant olfactory dysfunction, but these findings did not correlate with neuropsychiatric symptoms, biomarkers or brain structural abnormalities	9	GP patients exhibited olfactory dysfunction, with odour identification positively correlating with cognitive function
Lu *et al*. [34]	To assess grey matter microstructure in patients with early-stage neurosyphilis using voxel and surface-based morphometry analyses	Penicillin	Multiple areas with decreased grey matter volumes were identified, such as within the left frontal cortices and bilateral temporal/occipital cortices. There was significantly reduced cortical thickness in the right medial orbitofrontal lobe and reduced gyrification index in the left insula. Grey matter volumes in the middle frontal gyrus, gyrification index in the left insula and cortical thickness in the right medial orbitofrontal lobe were negatively correlated to the scores of a psychomotor speed and neurological dysfunction test (number connection test A)	8	In NS patients with no conventional MRI abnormality, changes in grey matter volume reduction and other morphological changes could be related to cognitive impairment
Luo *et al*. [40]	To assess and compare the biomarker levels in GP and ANS with AD and normal controls	No information	CSF levels of Aβ42 were significantly different between GP, ANS, control and AD groups, with GP being lower compared to ANS and controls; however, CSF A*β*40 was not. AD patients had higher CSF t-tau and p-tau181 levels than all other groups	7	Biomarker levels differ between GP and AD patients
Paraskevas *et al*. [33]	To assess the levels of tau protein in different stages of syphilis compared to those with AD	Penicillin	MMSE scores were comparable between AD and NS patients, but these did not correlate with CSF total tau. Tau was increased in NS and in AD when compared to controls and syphilis, which had not progressed to nervous system involvement	7	Total tau is increased in NS and may be used to discriminate from syphilis without nervous system involvement
Tong *et al*. [42]	To assess the clinical and laboratory features of GP and non-NS dementia	No information	Between the two groups, there were significantly different levels of total cholesterol, low-density lipoprotein cholesterol and homocysteine. CSF pleocytosis rates and increased protein levels were increased in the GP group	6	Patients with signs of dementia should undergo blood tests for syphilis
Wang *et al*. [35]	To compare the clinical and cognitive features of patients with GP to those with AD, FTD and controls	No information	Mild GP exhibited cognitive impairments in memory, language and executive function similar to mild AD, but showed mixed differences compared to mild FTD. GP patients reported more complaints and displayed more neurological signs than those with mild AD, and cranial MRI typically revealed medial temporal lobe atrophy	8	Cognitive decline in GP patients is comparable to that of patients with AD
Wang *et al*. [36]	To investigate the differing features of general paresis when compared to other dementias	Treated after baseline measurements taken	When compared with 47 healthy control participants regarding brain structure, GP patients had a greater volume loss in orbitofrontal, temporal, precuneus, insula and hippocampus areas. Whole-brain atrophy was frequently observed. GP can be distinguished from AD and FTD due to the presence of psychiatric symptoms such as delusions and irritability	6	Psychiatric symptoms differ between GP and AD patients
Zhang *et al*. [43]	To assess and compare the biomarker levels and cognitive function in NS and GP compared to those with AD	No information	CSF and plasma BACE1, alongside CSF tau and neurogranin, were all significantly higher in AD than in GPI. In mild stages of cognitive impairment, biomarker levels differed between the groups. Levels of CSF tau and plasma neurogranin correlated with cognitive scale scores in pooled NS scores	8	Biomarker levels differ between NS and AD patients
Zhong *et al*. [41]	To assess the neuropsychiatric features of NS compared to those with AD	Psychotropic drugs, antibiotics not stated	NS-MCI patients displayed significantly higher scores testing for psychosis and psychomotor syndromes than the controls, with NS-dementia scoring higher on affective and frontal syndromes in addition to psychosis and psychomotor syndromes. Frontal lobe syndrome was significantly more severe in patients with NS than in patients with AD from MCI through to moderate dementia stages	8	NS patients display different patterns of psychiatric symptoms than those with AD at the MCI and dementia stages

AD, Alzheimer’s disease; AI, antibody index; ANS, asymptomatic neurosyphilis; Aβ, amyloid beta; BACE1, Beta-site APP Cleaving Enzyme 1; FTD, frontotemporal dementia; GP, general paresis; NOS score, NOS risk of bias score; NS, neurosyphilis; NS-MCI, neurosyphilis with mild cognitive impairment; p-tau181, Phosphorylated Tau at Threonine 181; SF-36, short form health survey.

**Fig. 3.**
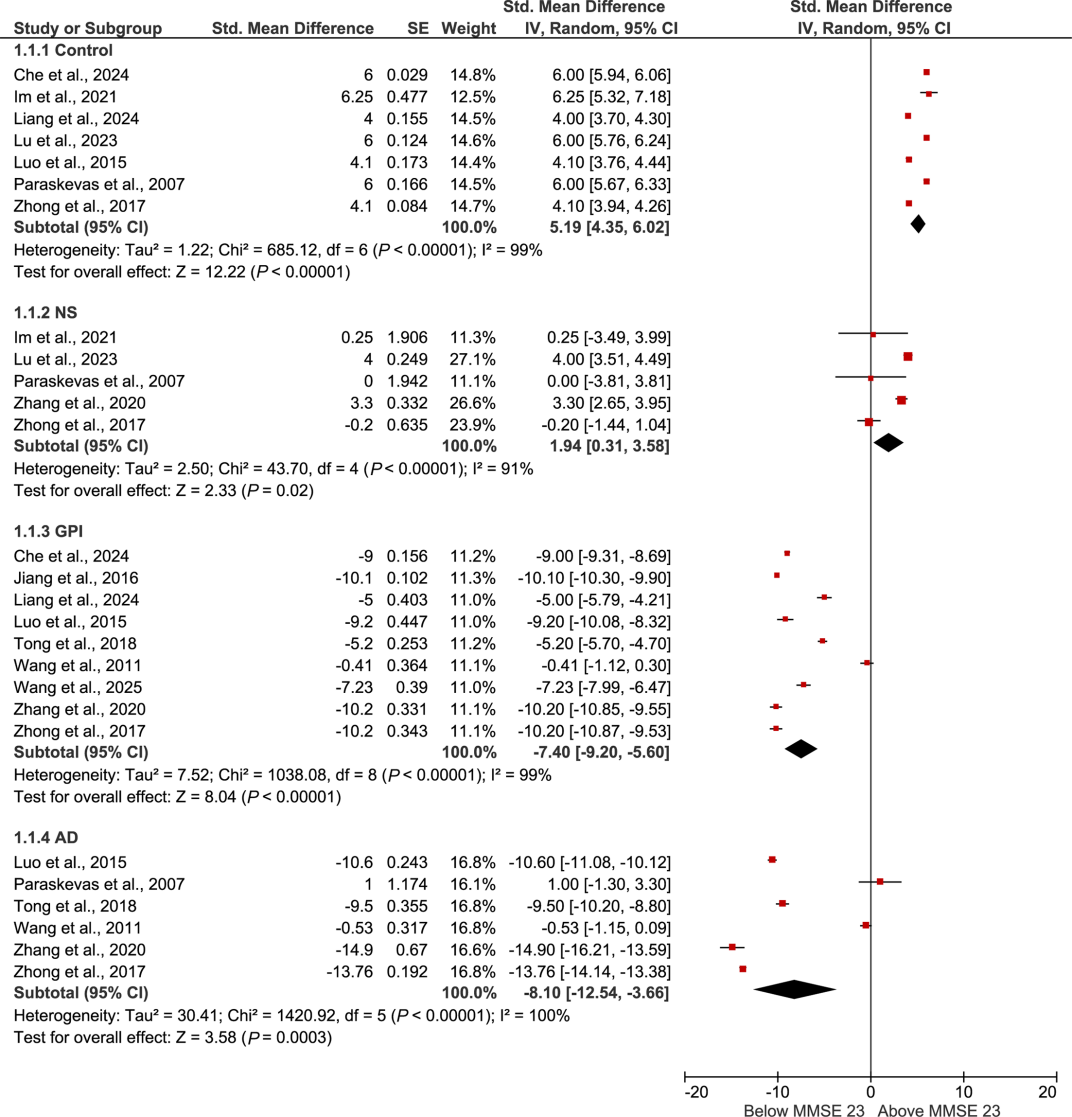
Forest plot of MMSE scores centred on the cognitive-impairment threshold (MMSE=23). Study-level mean scores were transformed by subtracting 23, such that 0 denotes the impairment threshold, negative values indicate impaired performance and positive values indicate scores above the threshold. Centred means and corresponding standard errors (calculated from reported standard deviations and sample sizes) were entered into RevMan 5.3 using the generic inverse variance method. Subgroup-level pooled estimates are presented for each diagnostic category [controls, neurosyphilis (NS), general paresis (GPI) and Alzheimer’s disease (AD)]. Error bars represent 95 % confidence intervals, and black diamonds indicate the pooled estimate for each subgroup.

On an MMSE scale centred at the cognitive impairment threshold (MMSE=23), the synthesized MMSE data from syphilis studies demonstrated clear separation between diagnostic groups (Fig. 3). Control participants consistently scored well above the impairment threshold (mean centred scores ranging from +4.36 to +6.02), indicating intact cognition. Patients with neurosyphilis scored only slightly above the threshold (+1.94), consistent with mild impairment. In contrast, individuals with general paresis (–7.40; 95 % confidence interval (CI) –9.20 to –5.60) and those with Alzheimer’s disease (–8.10; 95 % CI –12.54 to –3.66) showed marked cognitive deficits well below the impairment threshold.

### Cognitive and neurological outcomes following *B. burgdorferi* infection

**Fig. 4.**
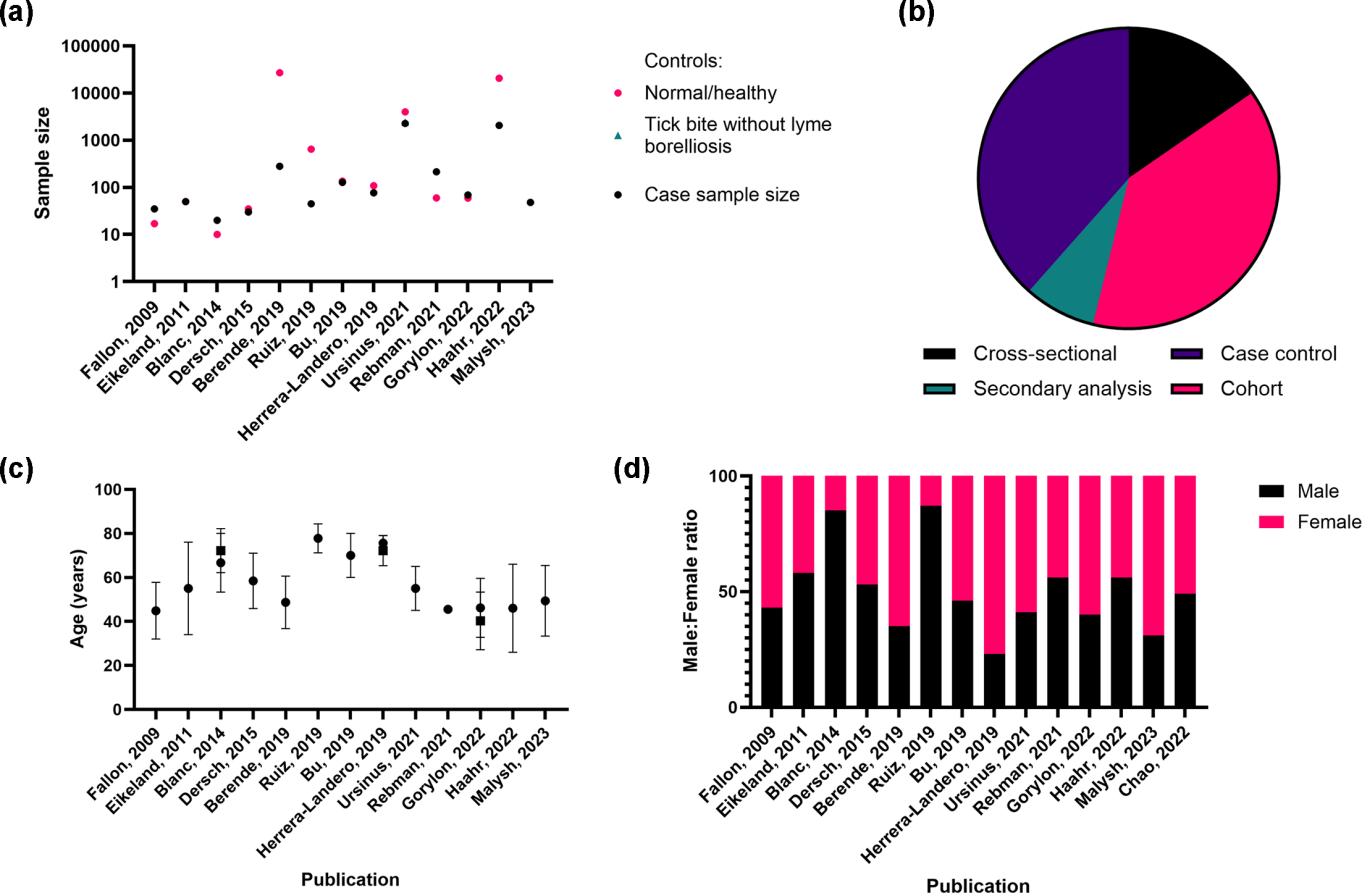
Description of population by study for Lyme disease. (**a**) Comparison of sample sizes for cases vs. controls, (**b**) number of publications by study design, (**c**) mean age of participants in the studies (±sd) and (**d**) ratio of male to female participants per study.

Thirteen studies investigating the cognitive and neurological outcomes associated with *B. burgdorferi* infection were included in this review (Fig. 4 and Table S6). These studies were conducted across a diverse range of countries, including the USA (*n*=3), France (*n*=2), the Netherlands (*n*=2) and one study each from Ukraine, Mexico, Germany, China, Norway and Denmark. The majority of studies focused on the association between post-treatment Lyme disease syndrome (4/13) or Lyme neuroborreliosis (3/13) and persistent cognitive deficits or neurodegenerative outcomes. Studies also examined the impact of these conditions on patients’ quality of life, highlighting long-term neurological and functional impairments following infection.

Of the 13 studies on *B. burgdorferi*, findings were heterogenous. Five studies reported no long-term cognitive impairment following treatment for Lyme neuroborreliosis, while the others identified persistent symptoms or increased Alzheimer’s disease risk. Neuroimaging findings included frontal hypometabolism, focal atrophy and reduced cerebral blood flow in temporal, parietal and limbic regions.

Neuroimaging was employed in two studies, using positron emission tomography (PET), fluorodeoxyglucose PET and MRI [46,47]. Fallon *et al*. reported reduced regional cerebral blood flow and cerebral metabolic rate in the parietal, temporal and limbic regions of the brain among patients with persistent Lyme encephalopathy [47]. Another study observed increased focal brain atrophy and frontal hypometabolism in patients with neurodegenerative dementia and a positive ‘intrathecal anti-*Borrelia* antibody index’, compared to those with neuroborreliosis and dementia, with no worsening observed following treatment [46].

Regarding cognitive outcomes, 5/13 (38 %) studies reported no long-term objective cognitive impairment after treatment, with patients treated for Lyme neuroborreliosis performing comparably to controls [48,52]. Of these five studies, 3/5 (60 %) still noted reduced quality of life in a subset of patients despite the absence of measurable cognitive deficits [48,50]. One study reported a transient increase in the risk of Guillain–Barré syndrome within the first-year post-treatment, which was not sustained beyond that period [51].

In contrast, two studies reported an increased prevalence of persistent symptoms, including reduced physical health scores and impaired social, emotional and physical functioning [53,54]. Herrera–Landero *et al*. found that *Borrelia* sp. seropositivity was associated with an increased risk of Alzheimer’s disease [adjusted odds ratio (aOR) = 3.65; 95 % CI 1.2–11.1] and mild cognitive impairment (aOR=3.2; 95 % CI 1.1–9.1) [55]. Another study examining overall infectious burden also reported a trend toward increased Alzheimer’s disease risk in those seropositive for *B. burgdorferi* (aOR=2.006 (95 % CI 0.879–4.576) [56]. Additional studies identified specific cognitive deficits, including impairments in language fluency and contrast sensitivity, in subsets of patients with prior Lyme disease [54,57]. The objectives, methodologies and key findings of these studies are summarized in Tables 2 and S7.

**Table 2.** Description of objectives, treatments and main outcomes by study (Lyme disease)

Author	Objective	Treatment	Main outcome	NOS score	Overview
Berende *et al*. [48]	To determine if the persistent symptoms attributed to Lyme borreliosis are validated by objective cognitive testing	Not required	The majority of patients (85.4%) displayed sufficient performance validity, of which 2.9 % were classified as cognitively impaired. There was no association between subjective cognitive symptoms and objective impairment	6	Only a small proportion of patients with persistent symptoms attributed to borreliosis exhibit objective cognitive impairment
Blanc *et al*. [46]	To prospectively study patients with dementia and a positive intrathecal anti-Borrelia antibody index (AI)	Ceftriaxone	Two groups were identified, both with comparable cognitive scores at baseline measurement; one with neuroborreliosis and an improvement of dementia after antibiotic treatment, and the other with progressive worsening of dementia symptoms. In this second group, final diagnoses included AD, Lewy body disease and others. Biomarker concentrations, such as tau, p-tau and A*β*42, in CSF in the second group were also consistent with the respective diagnoses, but normal in the neuroborreliosis group	5	Isolated Lyme dementia has a good outcome after antibiotics, but positive AI can also present in conjunction with neurodegenerative diseases, which may play a role in disease pathogenesis
Bu *et al*. [56]	To examine the association between infectious burden (via seropositivity) and Alzheimer’s disease	No information	In the case of single seropositivity, *B. burgdorferi* exhibited a significant association with AD (OR: 2.006). Elderly people with a higher infectious burden and seropositivity to four to five pathogens have a significantly higher amount of inflammatory cytokines and serum beta-amyloid protein levels compared to those who had been exposed to zero to three of the selected pathogens	7	There was a significant association with seropositivity to *B. burgdorferi* and AD. This supports the role of inflammation in the cause and pathogenesis
Dersch *et al*. [49]	To compare quality of life, fatigue, depression, cognitive function and verbal learning between patients with definite LNB and healthy controls	Ceftriaxone, doxycycline or both antibiotics	Initial presentations of LNB varied, but no significant differences were observed between patients and healthy controls regarding fatigue, quality of life, depression, verbal memory or cognitive impairment. However, patients with residual symptoms reported significantly lower quality of life scores compared to those without residual symptoms	7	Patients who had been treated for LNB do not commonly experience residual cognitive symptoms, but some may experience a lower quality of life
Eikeland *et al*. [50]	To compare the neuropsychological functioning in patients with Lyme neuroborreliosis 30 months after treatment to matched controls	Ceftriaxone and doxycycline	All participants displayed adequate test effort. Treated LNB patients scored lower than controls on tasks assessing attention/executive function, processing speed, visual memory and verbal memory. While the proportion of patients and controls with normal or mild deficits was similar, significantly more LNB-treated patients had scores indicating severe impairment (>2 sd below the mean) compared to controls (8 vs. 1, *P*=0.014).	8	Patients who had been treated for LNB performed comparably to controls, with a small subset experiencing debilitating cognitive problems
Fallon *et al*. [47]	To assess whether patients with Lyme disease and persistent cognitive deficits exhibit global or regional abnormalities in cerebral blood flow or metabolic rate	Cephalosporin	Consistent abnormalities were found in both regional cerebral blood flow and metabolic rate, which reflected hypoactivity. Affected regions included bilateral grey and white regions; primarily the temporal, parietal and limbic areas. Close coupling between blood flow and metabolic rate suggests a metabolically driven cause of these changes. When resting, the measurements for the two groups were not significantly different between patients and controls	7	Patients who experienced continued memory impairment after treatment for Lyme disease had objectively quantifiable topographic abnormalities in functional brain activity
Gorlyn *et al*. [57]	To investigate language fluency deficits as a secondary or independent deficit in PTLD	Prior intravenous and oral antibiotic treatment	Both the PTLD and MDD groups showed poorer verbal fluency than healthy controls in unadjusted analyses. However, in PTLD patients, language fluency deficits persisted even after accounting for verbal ability, slowed processing speed and memory difficulties, indicating a specific impairment beyond these factors	9	Language fluency may be a separate and important area of neurocognitive deficit in PTLD
Haahr *et al*. [51]	To examine the associations between LNB and dementia, AD, Parkinson’s disease, motor neuron disease, epilepsy and Guillain–Barré syndrome	No information	Long-term risks of Alzheimer’s disease, Parkinson’s disease, motor neurone disease, epilepsy or dementia were not observed. Within the first year after LNB diagnosis, however, there was a small risk of patients being diagnosed with Guillain–Barré syndrome when compared to the controls. This risk was not sustained past the initial year	9	No long-term risks of neurodegenerative diseases or other neurological conditions were identified after LNB diagnosis; however, a short-term increased risk of Guillain–Barré syndrome
Herrera-Landero *et al*. [55]	To determine the association of *B. burgdorferi* infection with AD or MCI in older adults	No information	In 38 patients with AD, 11 (29 %) had a positive IgG serology to *B. burgdorferi*. In 39 with MCI, 9 (23 %) had a positive serology, alongside 11 out of 108 controls (10%). For those with AD, this is an estimated adjusted odds ratio of 3.65	8	An increased risk of AD and MCI in seropositive patients to *B. burgdorferi* was observed
Malysh *et al*. [53]	To assess the cognitive functioning and quality of life of patients with LB	No information	Patients with Lyme borreliosis experienced significantly lower scores for physical health and reduced social and emotional functioning when compared to healthy controls. 64.6 % of the total LB patients were found to have cognitive impairment when assessed using the MoCA. This deterioration was strongly influenced by disease characteristics such as duration, the presence of Lyme arthritis and disease stage, such as neuroborreliosis	7	LB was observed to have a significant impact on the quality of life of patients, including cognitive impairment in the majority
Rebman *et al*. [54]	To investigate whether contrast sensitivity (the ability to discern sharp and clear outlines in the shading of objects) is altered in PTLD patients, and if this is associated with cognitive deficits	Yes, antibiotic	Contrast sensitivity scores did not differ overall; PTLD patients were nearly twice as likely to have contrast sensitivity impairment in either eye, which significantly increased the odds of belonging to the PTLD group (OR=2.6). Within PTLD, contrast sensitivity impairment was not linked to ocular complaints but was associated with neurologic abnormalities and showed a borderline association with cognitive impairment	7	In PTLD patients, contrast sensitivity is significant and linked to signs of cognitive and neurologic impairment
Ruiz *et al*. [52]	To investigate the association with seropositivity to *B. burgdorferi* and incidental neuropsychiatric disorders and functional decline	No information	The seroprevalence of *B. burgdorferi* rate was 6.5 %(45/689) among a group of older adults living in a rural environment in southwestern France. No increased risk of any of the studied outcomes of interest (cognitive decline, depressive symptoms and disability) was found, either in crude analysis or after adjustment	8	Seropositivity to *B. burgdorferi* does not appear to be a risk factor for incidental neuropsychiatric disorders and functional decline
Ursinus *et al*. [59]	To assess the prevalence and severity of persistent symptoms attributed to LB	Doxycycline, amoxicillin, azithromycin, ceftriaxone or other antibiotics	When compared to population and tick bite cohorts, the prevalence of persistent symptoms in LB patients was significantly higher. After a year, fatigue, cognitive impairment and pain were more severe in erythema migrans patients when compared to disseminated LB, in which pain was more severe. Background prevalence was substantial	7	In PTLD patients, persistent symptoms were significantly more prevalent and severe when compared to the population and tick bite cohorts

AD, Alzheimer’s disease; AI, antibody index; CSF, cerebrospinal fluid; CT, computed tomography; FTD, frontotemporal dementia; LB, Lyme Borreliosis; LNB, Lyme Neuroborreliosis; MCI, mild cognitive impairment; MDD, major depressive disorder; NOS score, NOS risk of bias score; PTLD, post-treatment lyme disease; SPECT, single-photon emission computed tomography.

**Fig. 5.**
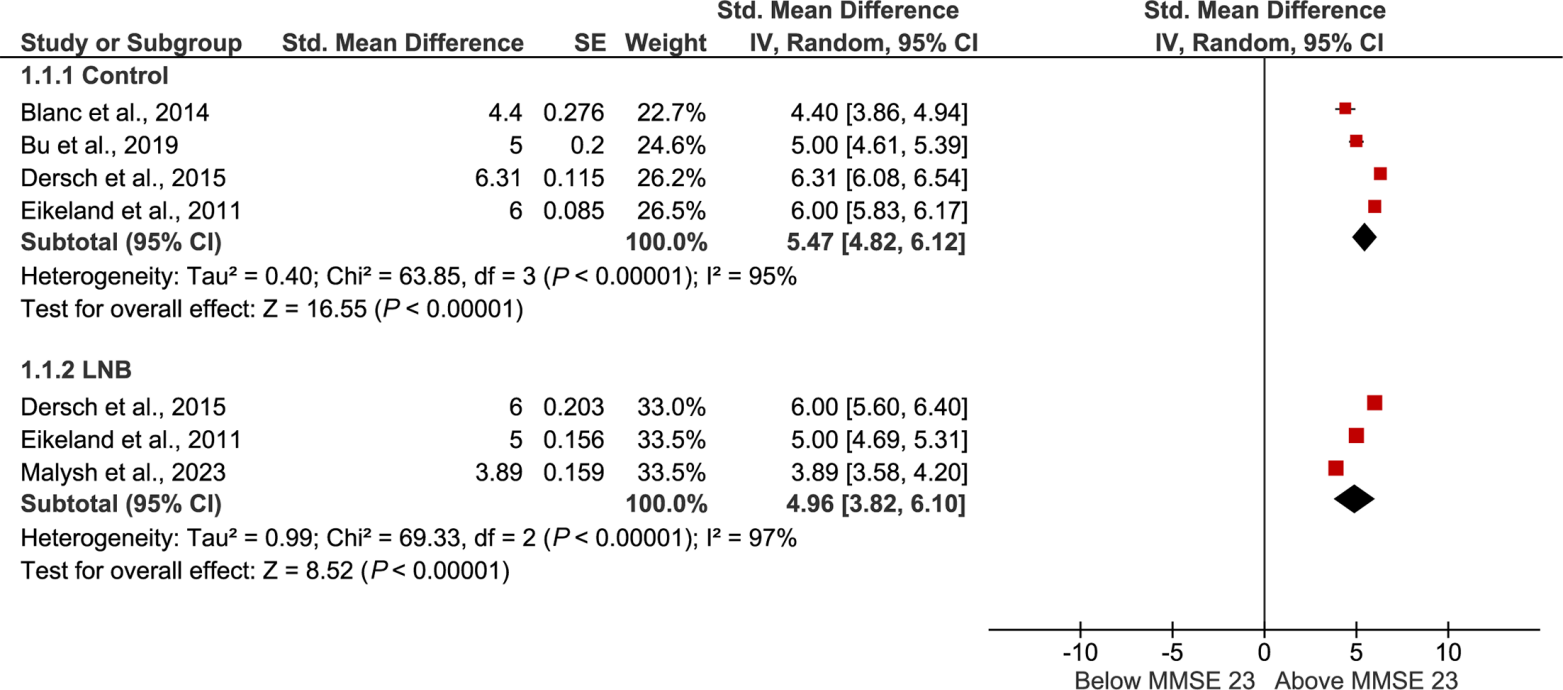
Forest plot of MMSE scores from Lyme disease studies, centred on the cognitive-impairment threshold (MMSE=23). Study-level mean scores were transformed by subtracting 23, such that 0 denotes the impairment threshold, negative values indicate impaired performance and positive values indicate scores above the threshold. Centred means and corresponding standard errors (calculated from reported standard deviations and sample sizes) were entered into RevMan 5.3 using the generic inverse variance method. Subgroup-level pooled estimates are presented for each diagnostic category [controls and Lyme neuroborreliosis (LNB)]. Error bars represent 95 % confidence intervals, and black diamonds indicate the pooled estimate for each subgroup.

For the MMSE scores extracted from the Lyme disease studies, control groups scored well above the cognitive-impairment threshold, with a pooled centred mean of +5.47 (Fig. 5). Patients with Lyme neuroborreliosis also plotted above the impairment threshold, showing scores broadly comparable to healthy controls (pooled centred mean of +4.96). The difference between the two groups was small, with Lyme neuroborreliosis patients scoring on average 0.51 points lower than controls.

### Evidence linking *Leptospira spp*. infection to neurodegenerative disease

One study investigating the association between leptospirosis and neurodegenerative diseases met the inclusion criteria for this review [58]. This retrospective matched cohort study from Taiwan included 357 patients with laboratory-confirmed leptospirosis and 1,071 age- and sex-matched controls. Over a 16-year follow-up period, leptospirosis was associated with a significantly increased risk of developing dementia (sub-distribution hazard ratio, 1.357; 95 % CI 1.213–1.519). Notably, antibiotic treatment, specifically *β*-lactams, cephalosporins or doxycycline, was associated with a reduced risk of dementia (Table S8).

## Discussion

This systematic review is the first to comprehensively evaluate observational evidence on the association between spirochaetal infections, specifically *T. pallidum*, *B. burgdorferi* and *Leptospira* spp. and cognitive impairment or neurodegenerative disease. Across 27 studies meeting inclusion criteria, consistent associations between syphilis and leptospirosis with cognitive decline were observed, while evidence linking Lyme disease to neurodegeneration was heterogeneous.

### Cognitive outcomes and neurodegenerative associations

Studies investigating syphilis and leptospirosis consistently reported cognitive impairment, often meeting diagnostic criteria for dementia. In contrast, findings related to Lyme disease varied widely. While some studies reported persistent cognitive symptoms [46,48, 50, 53, 59] or increased risk of Alzheimer’s disease [55,56], others found no significant long-term cognitive deficits following treatment for Lyme neuroborreliosis [49,51, 52]. Further, some studies reported persistent post-treatment symptoms or domain-specific cognitive deficits in subsets of patients [47,54, 57]. This inconsistency may reflect differences in diagnostic criteria, disease staging and methodological quality and underscores the need for longitudinal studies employing standardized diagnostic frameworks to clarify the temporal relationship between infection and neurodegenerative outcomes. Diagnostic accuracy remains a critical challenge; for example, enzyme immunoassays for *B. burgdorferi* demonstrate limited sensitivity, which may lead to underdiagnosis and consequently overestimate the proportion of patients progressing to neurodegenerative outcomes, as highlighted in systematic reviews on test performance and unconventional diagnostics [60,61].

Validated cognitive assessments such as MMSE, which can assess moderate to severe cognitive impairment [62], Clinical Dementia Rating, which assesses dementia of the Alzheimer type [63], and MoCA, which is suited to mild cognitive impairment [64], were commonly used. Though their diagnostic specificity for neurodegenerative disease is limited, they are an important predictor of conversion to dementia or similar impairment (Tables S5 and S7) [65]. Several studies employed formal diagnostic criteria (Diagnostic and Statistical Manual of Mental Disorders, International Classification of Diseases, National Institute of Neurological and Communicative Disorders and Stroke and the Alzheimer’s Disease and Related Disorders Association criteria [19,66, 67]), strengthening the reliability of reported outcomes. However, variability in the classification of both infection and cognitive impairment across studies continued to limit the extent and precision of direct comparisons, meaning that pooled subgroup estimates should be interpreted as descriptive rather than definitive.

The quantitative synthesis of MMSE data offers additional insight into these cognitive patterns across infection types. In the syphilis studies, centred MMSE scores demonstrated progressive cognitive decline consistent with disease stage: neurosyphilis patients showed mild impairment, whereas individuals with general paresis exhibited substantial deficits approaching those observed in Alzheimer’s disease. This aligns with previous evidence reporting an Alzheimer-like cognitive profile in mild general paresis. In contrast, pooled MMSE scores from Lyme neuroborreliosis studies indicated largely preserved global cognition, with treated patients performing close to healthy controls.

Together, these patterns reinforce the broader narrative emerging from the qualitative findings: cognitive impairment in syphilis is both common and clinically significant, while cognitive changes following Lyme disease are heterogeneous and, in many cases, minimal. However, the MMSE-based subgroup estimates should be interpreted cautiously, given substantial heterogeneity in diagnostic criteria, illness duration, treatment status and demographic characteristics across studies. Consequently, the pooled means provide descriptive summaries of general trends rather than precise comparative estimates.

### Neuroimaging manifestations of spirochaetal infections

The most consistent neuroimaging findings in diseases such as Alzheimer’s include cortical atrophy, especially in the medial temporal lobes and parietal–temporal areas, which is typically bilateral and symmetrical. A key finding is also the presence of A*β* plaques and tau tangles, which can also be visualized through PET scans. Patterns of Alzheimer’s disease on SPECT or PET images are characterized by bilateral hypoperfusion in the posterior parietal, temporal and posterior cingulate cortex with a lower degree of frontal involvement [68].

Within this review, six studies in total used a range of neuroimaging techniques. A pilot SPECT study found multifocal hypoperfusion in frontal, insular and posterior cingulate regions of those with neurosyphilis; however, this study had a small sample size (*n*=4), and all patients were male [37]. These results agree with earlier reports indicating reduced regional cerebral blood flow in the frontal regions; however, much of the existing evidence comes from case reports, which may employ non-standardized methods [69,71]. Atrophy and reduced metabolism of the posterior cingulate cortex are also observed in Alzheimer’s disease [72]. The involvement of the frontal cortex is less common in Alzheimer’s disease but is shown in frontotemporal dementia, which leads to problems with personality and social behaviour, often found in advanced neurosyphilis [34,37, 73]. Despite this, an included study noted that mild general paresis showed a pattern of cognitive impairment that was more consistent with Alzheimer’s disease than frontotemporal dementia, with medial temporal lobe atrophy similar to the presentation of Alzheimer’s disease [35]. In early neurosyphilis, without conventional MRI abnormality, grey matter reductions in frontal and temporal cortices were consistent with previous studies but could also benefit from validation with a larger cohort [34].

Two included studies investigated the neuroimaging characteristics of Lyme patients [46,47]. Fallon *et al.* [47] reported that in cases of well-documented and subsequently treated cases of Lyme disease, there was an objective topological change in functional brain activity, with regional cerebral blood flow and metabolic rate measurements of temporal, parietal and limbic regions [47]. The coupling between the two measurements suggests that the abnormalities observed are metabolically driven. The second study found there was more focal atrophy and frontal hypometabolism in a group of patients with a positive serology for * B. burgdorferi* and worsening after treatment; however, it cannot be excluded that these patients had pre-existing dementia [46]. A large proportion of the neuroimaging findings from Lyme disease patients are from case reports, with conclusions of nonspecific imaging features that vary between patients [74].

Taken together, these findings indicate that spirochaete-associated imaging abnormalities often differ from the canonical patterns of major neurodegenerative diseases. Neurosyphilis more frequently presents with frontal, insular and cingulate hypoperfusion or atrophy, whereas Alzheimer’s disease is characterized by bilateral medial temporal and parietotemporal involvement. Similarly, focal frontal hypometabolism observed in some Lyme-associated cases diverges from the typical posterior-predominant Alzheimer’s pattern. These distinctions suggest that while overlap exists, spirochaetal infections may produce neuroimaging signatures with different regional emphases compared with primary neurodegenerative disorders.

These studies demonstrate that neuroimaging findings vary between patients, disease and stages of disease. The studies presented cannot address whether any imaging abnormalities reflect persistent infection rather than a post-infectious process or are due to other common comorbidities such as depression [75].

### Biomarkers and pathophysiological insights

Several studies explored biomarkers associated with neurodegeneration in the context of spirochaetal infection. In neurosyphilis, elevated CSF tau and altered amyloid profiles were observed, though typically at lower levels than in Alzheimer’s disease. Increased neurogranin, BACE1 and CXCL13 were also reported, suggesting neuroinflammatory and metabolic involvement. However, biomarker data were limited and varied in quality, precluding definitive conclusions and underscoring the need for further research to clarify their diagnostic and prognostic value. Beyond biomarker profiles, the mechanisms by which spirochaetes invade the central nervous system and trigger neuroinflammation remain poorly understood. Proposed pathways include disruption of the blood-brain barrier, immune-mediated injury and persistent low-grade infection, but these hypotheses require validation in longitudinal and mechanistic studies [11,76].

### Diagnostic challenges and misclassification

The inclusion criteria for the diagnosis of spirochaetal infections within the present review were rigorous; only those studies with laboratory-confirmed diagnosis or use of appropriate serological tests, which indicated that probable exposure to *B. burgdorferi* or *T. pallidum* were included. Nevertheless, the inherent challenges in achieving accurate diagnosis highlight a critical area for future research. In healthcare settings, overdiagnosis of Lyme disease, often due to misinterpretation of serological tests, may inflate associations with cognitive decline [77].

Conversely, syphilis is often underdiagnosed due to its protean manifestations. These diagnostic issues underscore the need for standardized testing protocols and clinician education to avoid misclassification and inappropriate treatment.

### Strengths and limitations

The primary strength of this review lies in its systematic approach and strict inclusion criteria, ensuring that only studies with robust diagnostic and cognitive assessments were included, thereby reducing the risk of bias and reducing spurious associations between infection and neurodegenerative outcomes. However, the review is limited by the heterogeneity of study designs, small sample sizes and inconsistent reporting of confounders and treatment history. Although a quantitative synthesis using meta-analytic methods was possible for MMSE scores, substantial heterogeneity across the broader evidence base precluded calculation of an overall pooled estimate or quantitative synthesis for other cognitive or neurodegenerative outcomes. In many studies, incompatible diagnostic criteria, non-standardized cognitive assessments and the absence of extractable effect estimates meant that narrative synthesis remained the most appropriate approach for the majority of outcomes. It should also be noted that, because all available *T. denticola* studies were confounded by periodontitis and, therefore, excluded, this review does not conclude oral spirochaetes. The potential contribution of *T. denticola* to neuroinflammatory or neurodegenerative processes remains unresolved and requires studies that can disentangle organism-specific effects from the broader impact of periodontal disease.

## Conclusions

The findings highlight a potential role for spirochaetal infections in cognitive decline and neurodegeneration, particularly in syphilis and leptospirosis. However, the evidence for Lyme disease remains inconclusive. Future research should focus on longitudinal studies with standardized diagnostic criteria, integration of neuroimaging and biomarker data, exploration of mechanisms underlying central nervous system invasion and neuroinflammation and improved diagnostic testing and staging of infection.

Given rising global rates of syphilis, antimicrobial resistance and the potential impact of climate change on vector-borne diseases, understanding the neurological sequelae of spirochaetal infections is increasingly important, not only because dementia-related healthcare costs are projected to at least double by 2040 but also due to the profound personal and healthcare burden these conditions impose.

## Supplementary material

10.1099/jmm.0.002136 Uncited Supplementary Material 1.

10.1099/jmm.0.002136 Uncited Table S1.

## Abbreviations

aOR: adjusted odds ratio
apoA-I: apolipoprotein A-I
CNS: central nervous system
CSF: cerebrospinal fluid
MMSE: mini-mental state examination
MoCA: Montreal cognitive assessment
MRI: magnetic resonance imaging
NOS: Newcastle–Ottawa scale
PET: positron emission tomography
PRISMA: Preferred Reporting Items for Systematic Reviews and Meta-Analyses
SPECT: single-photon emission computed tomography
tau: tubulin associated unit
