# Supplementary material for: Association between spirochaetal infection and neurodegenerative diseases: a systematic review and quantitative synthesis of observational studies
Source: J Med Microbiol. 2026 Mar 6;75(3):002136. doi: 10.1099/jmm.0.002136 (PMC12967094; doi:10.1099/jmm.0.002136)
Supplement: Uncited Supplementary Material 1. [file jmm-75-02136-s001.pdf]

Supplementary information

Association between spirochaetal infection and neurodegenerative diseases: a systematic review and quantitative synthesis of observational studies

Mia Horton[1], Daniel J. Whiley[1,2], Megan Mayhew[1], and Samantha McLean[1]*

[1]School of Science and Technology, Nottingham Trent University, Clifton Lane, Nottingham, NG11 8NS, UK

[2]Medical Technologies Innovation Facility, Nottingham Trent University, Nottingham, United Kingdom.

*Corresponding author: School of Science and Technology, Nottingham Trent University, Clifton Lane, Nottingham, NG11 8NS, UK. E-mail address: samantha.mclean@ntu.ac.uk, tel: +44 (115) 8483324

Table S1 PRISMA checklist

PRISMA 2020 main checklist

Topic	No.	Item	Location where item is reported
**TITLE**			
**Title**	1	Identify the report as a systematic review.	Lines 1-3
**ABSTRACT**			
**Abstract**	2	See the PRISMA 2020 for Abstracts checklist	
**INTRODUCTION**			
**Rationale**	3	Describe the rationale for the review in the context of existing knowledge.	Lines 65-91
**Objectives**	4	Provide an explicit statement of the objective(s) or question(s) the review addresses.	Lines 92-98
**METHODS**			
**Eligibility criteria**	5	Specify the inclusion and exclusion criteria for the review and how studies were grouped for the syntheses.	Lines 105-137
**Information sources**	6	Specify all databases, registers, websites, organisations, reference lists and other sources searched or consulted to identify studies. Specify the date when each source was last searched or consulted.	Lines 138-145
**Search strategy**	7	Present the full search strategies for all databases, registers and websites, including any filters and limits used.	Supplementary table S2
**Selection process**	8	Specify the methods used to decide whether a study met the inclusion criteria of the review, including how many reviewers screened each record and each report retrieved, whether they worked independently, and if applicable, details of automation tools used in the process.	Lines 146-155
**Data collection process**	9	Specify the methods used to collect data from reports, including how many reviewers collected data from each report, whether they worked independently, any processes for obtaining or confirming data from study investigators, and if applicable, details of automation tools used in the process.	Lines 156-159
**Data items**	10a	List and define all outcomes for which data were sought. Specify whether all results that were compatible with each outcome domain in each study were sought (e.g. for all measures, time points, analyses), and if not, the methods used to decide which results to collect.	Lines 193-204
	10b	List and define all other variables for which data were sought (e.g. participant and intervention characteristics, funding sources). Describe any assumptions made about any missing or unclear information.	Lines 159-169

Topic	No.	Item	Location where item is reported
**Study risk of bias assessment**	11	Specify the methods used to assess risk of bias in the included studies, including details of the tool(s) used, how many reviewers assessed each study and whether they worked independently, and if applicable, details of automation tools used in the process.	Lines 184-191
**Effect measures**	12	Specify for each outcome the effect measure(s) (e.g. risk ratio, mean difference) used in the synthesis or presentation of results.	171-180
**Synthesis methods**	13a	Describe the processes used to decide which studies were eligible for each synthesis (e.g. tabulating the study intervention characteristics and comparing against the planned groups for each synthesis (item 5)).	171-183
	13b	Describe any methods required to prepare the data for presentation or synthesis, such as handling of missing summary statistics, or data conversions.	172-174
	13c	Describe any methods used to tabulate or visually display results of individual studies and syntheses.	177-179
	13d	Describe any methods used to synthesize results and provide a rationale for the choice(s). If meta-analysis was performed, describe the model(s), method(s) to identify the presence and extent of statistical heterogeneity, and software package(s) used.	171-177
	13e	Describe any methods used to explore possible causes of heterogeneity among study results (e.g. subgroup analysis, meta-regression).	178-180
	13f	Describe any sensitivity analyses conducted to assess robustness of the synthesized results.	Figs 3&5
**Reporting bias assessment**	14	Describe any methods used to assess risk of bias due to missing results in a synthesis (arising from reporting biases).	N/A
**Certainty assessment**	15	Describe any methods used to assess certainty (or confidence) in the body of evidence for an outcome.	Figs 3&5
**RESULTS**			
**Study selection**	16a	Describe the results of the search and selection process, from the number of records identified in the search to the number of studies included in the review, ideally using a flow diagram.	Lines 193-204, Figure 1
	16b	Cite studies that might appear to meet the inclusion criteria, but which were excluded, and explain why they were excluded.	Lines 209-213
**Study characteristics**	17	Cite each included study and present its characteristics.	Results section, (lines 192-316), tables 1 and 2
**Risk of bias in studies**	18	Present assessments of risk of bias for each included study.	Table 1, Table 2, Table S2

Topic	No.	Item	Location where item is reported
**Results of individual studies**	19	For all outcomes, present, for each study: (a) summary statistics for each group (where appropriate) and (b) an effect estimate and its precision (e.g. confidence/credible interval), ideally using structured tables or plots.	Figures 2-5, Table S4-S8
**Results of syntheses**	20a	For each synthesis, briefly summarise the characteristics and risk of bias among contributing studies.	Table S3, Figure 3, Figure 5
	20b	Present results of all statistical syntheses conducted. If meta-analysis was done, present for each the summary estimate and its precision (e.g. confidence/credible interval) and measures of statistical heterogeneity. If comparing groups, describe the direction of the effect.	Figure 3, Figure 5
	20c	Present results of all investigations of possible causes of heterogeneity among study results.	Figure 3, Figure 5
	20d	Present results of all sensitivity analyses conducted to assess the robustness of the synthesized results.	Figure 3, Figure 5
**Reporting biases**	21	Present assessments of risk of bias due to missing results (arising from reporting biases) for each synthesis assessed.	Table S3, Figure 3, Figure 5
**Certainty of evidence**	22	Present assessments of certainty (or confidence) in the body of evidence for each outcome assessed.	Figure 3, Figure 5
**DISCUSSION**			
**Discussion**	23a	Provide a general interpretation of the results in the context of other evidence.	Discussion section, Lines 355-502
	23b	Discuss any limitations of the evidence included in the review.	Lines 478-490
	23c	Discuss any limitations of the review processes used.	Lines 380-387
	23d	Discuss implications of the results for practice, policy, and future research.	Lines 494-497
**OTHER INFORMATION**			
**Registration and protocol**	24a	Provide registration information for the review, including register name and registration number, or state that the review was not registered.	Lines 101-104
	24b	Indicate where the review protocol can be accessed, or state that a protocol was not prepared.	Lines 103-104
	24c	Describe and explain any amendments to information provided at registration or in the protocol.	N/A
**Support**	25	Describe sources of financial or non-financial support for the review, and the role of the funders or sponsors in the review.	Lines 513-514
**Competing interests**	26	Declare any competing interests of review authors.	Lines 511-512

Topic	No.	Item	Location where item is reported
**Availability of data, code and other materials**	27	Report which of the following are publicly available and where they can be found: template data collection forms; data extracted from included studies; data used for all analyses; analytic code; any other materials used in the review.	Lines 103-104

From: Page MJ, McKenzie JE, Bossuyt PM, Boutron I, Hoffmann TC, Mulrow CD, et al. The PRISMA 2020 statement: an updated guideline for reporting systematic reviews. MetaArXiv. 2020, September 14. DOI: 10.31222/osf.io/v7gm2. For more information, visit: www.prisma-statement.org

Table S2 Search terms used in their corresponding database.

Database	Search terms	No. articles
Scopus	(ABS (((lyme) AND (disease OR neuroborreliosis)) OR borreli* OR spirochet* OR spirochaet* OR treponem* OR syphil is OR neurosyphilis OR leptospir*) AND ABS (neurodegener* OR neuro-degener* OR parkinson* OR alzheimer* OR dement* OR ((cognitive OR neurocognitive OR neuro-cognitive) AND (impairment* OR degenerati* OR disord*)))) AND (EXCLUDE (DOCTYPE , "re") OR EXCLUDE (DOCTYP E , "ch") OR EXCLUDE (DOCTYPE , "cp") OR EXCLUDE (DOCTYPE , "sh") OR EXCLUDE (DOCTYPE , "no") OR EXCLUDE (D OCTYPE , "le") OR EXCLUDE (DOCTYPE , "cr") OR EXCLUDE (DOCTYPE , "er") OR EXCLUDE (DOCTYPE , "ed") OR EXCLU DE (DOCTYPE , "bk"))	420
PubMed	((((lyme[Title/Abstract]) AND (disease[Title/Abstract] OR neuroborreliosis[Title/Abstract])) OR borreli*[Title/Abstract] OR spirochet*[Title/Abstract] OR spirochaet*[Title/Abstract] OR treponem*[Title/Abstract] OR syphilis[Title/Abstract] OR neurosyphilis[Title/Abstract] OR leptospir*[Title/Abstract]) AND (neurodegener*[Title/Abstract] OR neuro-degener*[Title/Abstract] OR parkinson*[Title/Abstract] OR alzheimer*[Title/Abstract] OR dement*[Title/Abstract] OR ((cognitive[Title/Abstract] OR neurocognitive[Title/Abstract] OR neuro-cognitive[Title/Abstract]) AND (impairment*[Title/Abstract] OR degenerati*[Title/Abstract] OR disord*[Title/Abstract]))) AND (2000/1/1:2025/5/6[pdat])) NOT (Review[Publication Type])	440
Web of Science	((AB=(((lyme) AND (disease OR neuroborreliosis)) OR borreli* OR spirochet* OR spirochaet* OR treponem* OR syphilis OR neurosyphilis OR leptospir*)) AND AB=(neurodegener* OR neuro-degener* OR parkinson* OR alzheimer* OR dement* OR ((cognitive OR neurocognitive OR neuro-cognitive) AND (impairment* OR degenerati* OR disord*)))) NOT DT=(Review)	358
EBSCOhost (includes CINAHL Ultimate and Academic Search Complete)	((lyme) AND (disease OR neuroborreliosis)) OR borreli* OR spirochet* OR spirochaet* OR treponem* OR syphilis OR neurosyphilis OR leptospir* AND neurodegener* OR neuro-degener* OR parkinson* OR alzheimer* OR dement* OR ((cognitive OR neurocognitive OR neuro-cognitive) AND (impairment* OR degenerati* OR disord*))	390

Table S3 Newcastle-Ottawa Scale risk of bias assessment

		Bacterial species																										
		Treponema pallidum													***Borrelia burgdorferi***													***L***
	Criteria for scoring ↓	Lu et al., (2023)	Zhang et al., (2020)	Paraskevas et al., (2007)	Wang et al., (2025)	Tong et al., (2018)	Luo et al., (2015)	Che et al., (2024)	Zhong et al., (2017)	Liang et al., (2024)	Gyanwali et al., (2018)	Im et al., (2021)	Jiang et al., (2016)	Wang et al., (2011)	Berende et al, (2019)	Blanc et al, (2014)	Bu et al, (2019)	Dersch et al, (2015)	Eikeland et al, (2011)	Fallon et al, (2009)	Gorlyn et al, (2022)	Haahr et al, (2020)	Herrera-Landero et al, (2019)	Malysh et al, (2023)	Rebman et al, (2021)	Ruiz et al, (2019)	Ursinus et al, (2021)	Chao et al, (2022)
**Selection**	1) Is the case definition adequate? a) yes, with independent validation* b) yes, e.g. record linkage or based on self-reports c) no description	a*	a*	a*	a*	a*	a*	a*	a*	a*	a*	a*	a*	a*	a*	a*	a*	a*	a*	a*	a*	a*	a*	a*	a*	a*	a*	a*
	2) Representativeness of the cases a) consecutive or obviously representative series of cases* b) potential for selection biases or not stated	b	a*	a*	a*	a*	a*	a*	a*	a*	b	b	a*	a*	a*	b	b	a*	b	a*	a*	a*	b	a*	b	b	a*	a*
	3) Selection of Controls a) community controls* b) hospital controls c) no description	a*	c	b	b	b	b	b	a*	a*	b	b	b	a*	c	a*	b	a*	a*	c	a*	a*	a*	c	a*	a*	a*	b
	4) Definition of Controls a) no history of disease (endpoint)* b) no description of source	a*	a*	b	a*	a*	a*	a*	a*	a*	a*	a*	a*	a*	b	a*	a*	a*	a*	a*	a*	a*	a*	a*	a*	a*	a*	a*
**Comparability**	1) Comparability of cases and controls on the basis of the design or analysis a) study controls for age* b) study controls for sex*	a* b*	a* b*	a* b*	a* b*	c	a*	c	a*	a* b*	a* b*	a* b*	c	a*	a* b*	c	a* b*	c	a* b*	a* b*	a* b*	a* b*	a* b*	a* b*	a*	a* b*	a* b*	a* b*
**Exposure**	1) Ascertainment of exposure a) secure record (e.g. surgical records)* b) structured interview where blind to case/control status* c) interview not blinded to case/control status d) written self-report or medical record only e) no description	a*	a*	a*	a*	a*	a*	a*	a*	a*	a*	a*	a*	a*	b*	a*	a*	b*	a*	a*	a*	a*	a*	b*	a*	a*	a*	a*
	2) Same method of ascertainment for cases and controls a) yes* b) no	a*	a*	a*	b	a*	a*	a*	a*	a*	a*	a*	a*	a*	b	b	a*	a*	a*	a*	a*	a*	a*	b	a*	a*	b	a*
	3) Non-Response rate a) same rate for both groups* b) non respondents described c) rate different and no designation	a*	a*	a*	b	a*	a*	a*	a*	a*	b	a*	b	a*	a*	a*	a*	a*	a*	c	a*	a*	a*	a*	a*	a*	b	a*
	**Total score**	**8**	**8**	**7**	**6**	**6**	**7**	**6**	**8**	**9**	**6**	**7**	**5**	**8**	**6**	**5**	**7**	**7**	**8**	**7**	**9**	**9**	**8**	**7**	**7**	**8**	**7**	**8**

L: *Leptospira spp.*

Table S4 Description of population by study (Syphilis)

First author (year)	Country	Study design	Duration of follow up	Age (mean ± SD/Range)	Sample size, cases (Male/Female)	Sample size, controls	Control matching	Funding source	Reference
Che et al., (2024)	China	Cross-sectional	N/A	GP 55.76 ± 9.15	GP 80 (68/12)	57 NC	Not stated	Natural Science Foundation of Guangdong Province, Guangzhou Municiple Health Commission, Plan on enhancing scientific research in GMU, Guangzhou Municipal Science and Technology Bureau, Guangzhou Municipal Key Discipline in Medicine, Guangzhou High-level Clinical Key Specialty, Guangzhou Research oriented Hospital, Guangzhou Municipal Science and Technology Bureau	[37]
Gyanwali et al., (2018)	Singapore	Retrospective cross-sectional	N/A	Demented memory clinic 76(10)	Demented memory clinic 745 (321/424)	526 NC (memory clinic)<br>872 NC (community-dwelling)	Not stated	National Medical Research Council	[44]
Im et al., (2021)	South Korea	Cross-sectional	N/A	NS 46.3 ± 7.6	NS 4 (4/0)	4 NC	Age and sex	Korea Health Industry Development Institute, Korea Dementia Research Center, and the National Research Foundation of Korea	[36]
Jiang et al., (2016)	China	Retrospective cross-sectional	3 months post admission	GP 52.3 ± 10.0, Syphilis 46.3 ± 14.2	GP 188 (165/23) syphilis 241 (135/106)	539 NC	Not stated	Israel Science Foundation, National Natural Science Foundation of China, Medical Research Foundation of Guangdong Province	[38]
Liang et al., (2024)	China	Cross-sectional	N/A	GP 55.5±10.3	40 GP (36/4)	37 HC	Age and sex	Not stated	[43]

Lu et al., (2023)	China	Cross-sectional	N/A	NS 47.84 ± 9.09	NS 19 (11/8)	19 HC	Age, sex, education	National Natural Science Foundation of China, Natural Science Foundation of Tianjin, Tianjin Key Medical Discipline (Specialty) Construction Project	[33]
Luo et al., (2015)	China	Cross-sectional	N/A	GP: 52.6 ± 9.3. ANS: 50.3 ± 13.3	GP 44 (38/ 6) ANS 10 (7/3)	36 NC 53 AD	Not stated	Chinese National Science Foundation, Guangdong Science and Technology Program	[39]
Paraskevas et al., (2007)	Greece	Cross-sectional	N/A	AD 51±7, NS 50±11, Syphilis 44±12	NS 12 (7/5), Syphilis 17 12(12/5)	13 NC with minor surgery 14 AD	Not stated	Not stated	[32]
Tong et al., (2018)	China	Retrospective comparative	N/A	GP 52 (47.0–61.0)	GP 85 (71/14)	196 Dementia	Not stated	National Natural Science Foundation, Key Projects for Province Science and Technology Program of Fujian, National Science Foundation for Distinguished Young Scholars of Fujian, Major Special Projects for Serious Illness of Xiamen, Natural Science Foundation of Fujian Province	[41]
Wang et al., (2011)	China	Cross-sectional	N/A	Mild GP 56.42 ± 9.50	Mild GP 12 (11/1)	36 NC 24 AD 11 FTD	Not stated	Natural Science Foundation of China, Science and Technology Commission of Shanghai Municipality	[34]
Wang et al., (2025)	China	Cohort	Mean of 1.4 years	GP 56.79 (10.87)	GP 78 (70/8)	47 HC 51 AD 32 FTD, 16 Anti-LGI1 encephalitis	Not stated	National Natural Science Foundation of China, Science and Technology Innovation 2030-Major Project for Brain Science and Brain-like Research	[35]
Zhang et al., (2020)	China	Cross-sectional	N/A	AD 65.9±8.9, GP 53.2±10.7, NS 46.4±11.4	AD 23 (8/15) GP 55 (49/6), NS 13 (10/3)	23 AD	Not stated	National Natural Science Foundation of China, Guangzhou Municipal Psychiatric Disease Clinical Transformation Laboratory, Science and	[42]

								Technology Plan Project of Guangdong Province, National Key Research and Development Program of China	
Zhong et al., (2017)	China	Case-control	N/A	NS-MCI 50.6±12.9, NS-dementia 52.6±10.2	NS-MCI 21 (15/16), NS-dementia 70 (60/10)	139 NC<br>162 AD<br>157 MCI	Not stated	National Natural Science Foundation of China, Guangzhou Municipal Science and Technology Program, Science and Technology Department of Guangdong Province major science and technology, National R&D program focused on precision medical research of China	[40]
	NC, normal control; *HC*, healthy control; AD, ; FTD, ; MCI,								

Table S5 Description of cognitive criteria, tests and/or diagnoses by study (Syphilis)

Paper	Che et al, (2024)	Gyanwali et al, (2010)	Im et al, (2021)	Jiang et al, (2016)	Liang et al, (2024)	Lu et al, (2023)	Luo et al, (2015)	Paraskevas et al, (2007)	Tong et al, (2018)	Wang et al, (2011)	Wang et al, (2025)	Zhang et al, (2020)	Zhong et al, (2017)
Diagnosis of syphilis, neurosyphilis or general paresis													
Name of test/diagnostic criteria ↓													
Rapid plasma reagin	Yes			CSF	Serum, CSF	Serum	Serum			Serum, CSF	Serum, CSF	Serum, CSF	Serum, CSF
Toluidine red unheated serum test	Yes												
T. pallidum hemagglutination test	Serum, CSF						Serum, CSF					Serum, CSF	Serum, CSF
White blood cell counts in CSF	10 x 10^6 L^{-1}		5 cells μL^{-1}	5 cells μL^{-1}			10 x 10^6 L^{-1}	Elevated	Elevated	10 x 10^6 L^{-1}	10 x 10^6 L^{-1}	10 x 10^6 L^{-1}	10 x 10^6 L^{-1}
Protein levels in CSF exceeding	500 mg L^{-1}		45 mg dl^{-1}				500 mg L^{-1}	Elevated	Elevated	500 mg L^{-1}	500 mg L^{-1}	500 mg L^{-1}	500 mg L^{-1}
Enzyme immunoassay		Yes											
Venereal Disease Research Laboratory test			CSF	CSF				Serum		CSF			
Fluorescent Treponemal Antibody Absorption test			Serum					Serum					
Otherwise unexplained neurological manifestation consistent with NS			Yes										Yes
T. pallidum particle agglutination test				CSF	Yes	Serum, CSF				Yes	Serum, CSF		

	Cognitive tests or diagnoses												
MMSE	X	X	X	X*	X*	X	X	X	X	X	X*	X	X*
Met DSM-IV criteria	X	X			X		X		X	X		X	
Clinical dementia rating			X				X		X		X	X	X*
Clinical dementia rating - sum of boxes			X										
Global deterioration scale			X										
Neuropsychiatric inventory (NPI)					X		X				X		
Number correction test A						X							
Digital symbol test						X	X	X					
Met NINCDS-ADRDA criteria											X	X	X
FTD diagnosed according to clinical consensus criteria										X			
Montreal cognitive assessment												X	
MCI diagnosed according to the Peterson criteria													X
Miscellaneous auditory and visual tests										X	X		

CSF, cerebrospinal fluid; DSM-IV, Diagnostic and Statistical Manual of Mental Disorders IV; FTD, frontotemporal dementia; MCI, mild cognitive impairment; MMSE, mini mental state examination; NINCDS-ADRDA, National Institute of Neurological and Communicative Disorders and Stroke and the Alzheimer's Disease and Related Disorders Association; NS, neurosyphilis.

Table S6 Description of population by study (Lyme disease)

Authors	Country	Study design	Duration of follow up (if applicable)	Age, cases (mean, SD/Range)	Sample size, cases (male/female)	Sample size, controls	Control matching	Funding source	Reference
Berende et al, (2019)	Netherlands	Cross-sectional	N/A	Persistent Lyme 48.7 (11.9)	Persistent Lyme 280 (151/279)	26,939 normative sample	Not stated	Netherlands Organisation for Health Research and Development	[47]
Blanc et al, (2014)	France	Prospective cohort	From between September 2005-September 2011 and June 2013.	NB dementia 66.7 ± 13.3 dementia and positive AI 72.2 ± 10.0	7 NB dementia (6/1), 13 NB dementia and positive AI (11/2)	10 NC	Not stated	Not stated	[45]
Bu et al, (2019)	China	Case-control	N/A	AD 70 (10)	AD 128 (59/69)	135 NC	Age, sex	National Natural Science Foundation of China, Natural Science Foundation Project of CQCSTC, the Army Health Care Project	[55]
Dersch et al, (2015)	Germany	Case-control	Follow up not stated	LNB 58.46 (12.6)	LNB 30 (16/14)	35 NC	Not stated	Not stated	[48]
Eikeland et al, (2011)	Norway	Longitudinal cohort	30 months	PTLD 55 (21 - 76)	PTLD 50 (29/21)	50 NC	age, gender, and education level	South-Eastern Health Authority of Norway	[49]
Fallon et al, (2009)	USA	Case-control	N/A	Persistent Lyme encephalopathy 44.9 (12.9)	Persistent Lyme encephalopathy 35 (15/20)	17 NC	Age, education and sex	National Institute of Neurological Disorders and Stroke, National Institute of Mental Health, Columbia University Lyme and Tick-borne Diseases Research Center, Lyme	[46]

								Disease Association, and Irving Institute for Clinical and Translational Research	
Gorlyn et al, (2022)	USA	Secondary analysis	N/A	PTLD 46.2 (13.4) MDD 40.3 (13.1)	PTLD 31 (14/17), MDD 38 (15/26)	59 NC	Not stated	Columbia University Medical Center, Steven & Alexandra Cohen Foundation, National Institute of Mental Health. National Institute of Neurological Disorders and Stroke	[56]
Haahr et al, (2020)	Denmark	Retrospective cohort	0-1 years, 1+ years	LNB 46 years (interquartile range, 12–62 years)	LNB 2067 (1163/904)	20,670 NC	Age and sex	Danish Council for Independent Research	[50]
Herrera-Landero et al, (2019)	Mexico	Case-control	N/A	AD 75.6(3.4), MCI 72.2(6.8)	AD 38 (12/26), MCI 39 (6/33),	108 NC	Age, sex. Ratio of 1:3 cases to controls	Instituto Mexicano del Seguro Social, Universidad Nacional Autónoma de Mexico	[54]
Malysh et al, (2023)	Ukraine	Case-control	N/A	LB 49.4 ± 16.03	LB 48 (15/33)	48 NC	Sex, age, nationality and place of birth	Not stated	[52]
Rebman et al, (2021)	USA	Cross-sectional	N/A	PTLD 45.5	PTLD 214 (119/95)	60 NC or recovered Lyme	Not stated	Steven and Alexandra Cohen Foundation	[53]
Ruiz et al, (2019)	France	Prospective cohort	2007 (baseline), 2010, 2013, 2016	Positive serology 77.8 (6.6)	Positive serology 45 (39/6)	644 negative serology	Not stated	AGRICA, la Mutualité Sociale Agricole de Gironde, la Caisse Centrale de la Mutualité Sociale Agricole	[51]

Ursinus et al, (2021)	Netherlands	Prospective cohort	3, 6, 9 and 12 months after inclusion	LB 55 (45–63)	Lyme patients 1135, EM 1076, disseminated 59 (465/670)	2405 tick bite without LB, 4000 NC	age, sex, geographical region, and month of enrolment	Netherlands Organization for Health Research and Development and Dutch Ministry of Health, Welfare and Sport	[58]

AD, Alzheimer's disease; AI, antibody index; EM, erythema migrans; LB, Lyme borreliosis; LNB, Lyme neuroborreliosis; MCI, mild cognitive impairment; MDD, major depressive disorder; NB, neuroborreliosis; NC, normal control; PTLD, post-treatment Lyme disease; SD, standard deviation.

Table S7 Description of cognitive criteria, tests and/or diagnoses by study (Lyme disease)

Paper	Berende et al., (2019)	Blanc et al., (2014)	Bu et al., (2019)	Dersch et al., (2015)	Eikeland et al., (2011)	Fallon et al., (2009)	Gorlyn et al., (2022)	Haahr et al., (2020)	Herrera-Landero et al., (2019)	Malysh et al., (2023)	Rebman et al., (2021)	Ruiz et al., (2019)	Ursinus et al., (2021)
Diagnosis of Lyme disease or Lyme seropositivity													
Name of test or diagnostic criteria ↓													
Presence of erythema migrans					Yes, in past 4 months		Inferred (IDSA)						Yes
Positive *B. burgdorferi* IgG or IgM antibodies	Yes			Serum CSF	Serum								Serum
ELISA		Yes	Yes			Inferred (CDC)	Inferred (IDSA)	Serum, CSF	IgG, serum	IgG&IgM serum CSF	Yes	IgG	
Positive intrathecal antibody index		Yes		Serum CSF	Yes			Definite if > 0.3					
Western blot		Serum CSF				IgG	IgG		Yes	IgG&IgM serum CSF	IgG&IgM	IgG	
Compatible neurological symptoms				Yes	Yes	Yes							
Lymphocytic pleocytosis				>5 leukocytes/ mm^3	>5 leukocytes/ mm^3								
Skin biopsy culture and PCR													Yes
Cognitive tests or diagnoses													
MMSE		X	X	X	X					X		X	
Met DSM-IV criteria		X										X	
Clinical dementia rating			X										

International Classification of Diseases - 10								X					
Met NINCDS-ADRDA criteria			X						X				
Montreal cognitive assessment									X	X			
Activities of Daily Living			X										
SF-36				X						X			X
Fatigue severity score				X	X								
Verbal learning memory test				X									
Beck's depression inventory				X			X						
Montgomery and Åsberg Depression Rating Scale					X								
Starkstein Apathy Scale					X								
Weschler Memory Scale						X							
Weschler Adult Intelligence Scale							X				X		
Checklist Individual Strength													X
Cognitive Failures Questionnaire													X
Battery of auditory and visual tests											X		
Battery of cognitive and neuropsychiatric tests	X	X				X	X				X	X	

ELISA, Enzyme linked immunosorbent assay; MMSE, Mini Mental State Examination; NINCDS-ADRDA, National Institute of Neurological and Communicative Disorders and Stroke and the Alzheimer's Disease and Related Disorders Association; PCR, Polymerase Chain Reaction; SF-36, 36-Item Short Form Survey; DSM-IV, Diagnostic and Statistical Manual of Mental Disorders IV.

Table S8 Characteristics of the included studies (Leptospirosis)

**Author**	Country	Study design	Duration of follow up (if applicable)	Age, cases (mean, SD/Range)	Sample size, cases (male/female)	Sample size, controls	Control matching	Funding source	Reference
Chao et al, (2022)	Taiwan	Retrospective matched cohort	16 years (2000-2016)	63.35 ± 8.68	357 Leptospirosis cohort (249/108)	1,071 Normal controls	Age, sex	Ministry of Defense of Taiwan, Tri-Service General Hospital Research Foundation, Taoyuan Armed Forces General Hospital	[55]

**Aim or objective of study**	**How was diagnosis performed**	**Antibiotic treatment**	**Cognitive tests or diagnoses**	**Qualitative outcome**
To explore the association between leptospirosis, the risk of dementia, and the potential protective role of antibiotic treatment.	Diagnosis performed according to the International Classification of Diseases, 9th Revision, Clinical Modification. Positive culture isolation, and/or a fourfold rise in microscopic agglutination test titre between the acute phase and the convalescent phase Titer ≥1:400 in a single serum.	Yes, β-lactams, cephalosporins, and doxycycline.	Performed by board-certified neurologists or psychiatrists according to the Diagnostic and Statistical Manual of Mental Disorders, 4th Edition and its text-revised edition.	Leptospirosis was associated with an increased risk for dementia, and antibiotic treatment was associated with a reduced risk.
